# 100- kyr cyclicity in volcanic ash emplacement: evidence from a 1.1 Myr tephra record from the NW Pacific

**DOI:** 10.1038/s41598-018-22595-0

**Published:** 2018-03-13

**Authors:** Julie C. Schindlbeck, Marion Jegen, Armin Freundt, Steffen Kutterolf, Susanne M. Straub, Maryline J. Mleneck-Vautravers, Jerry F. McManus

**Affiliations:** 1 GEOMAR Helmholtz Centre for Ocean Research Kiel, 24148 Kiel, Germany; 2 Institute of Earth Sciences, Heidelberg University, Im Neuenheimer Feld 234-236, 69120 Heidelberg, Germany; 3 Lamont-Doherty Earth Observatory of Columbia University, Palisades, New York, 10964 United States; 4 Department of Earth Sciences, University of Cambridge, Downing Street, Cambridge, CB2 3EQ United Kingdom

## Abstract

It is a longstanding observation that the frequency of volcanism periodically changes at times of global climate change. The existence of causal links between volcanism and Earth’s climate remains highly controversial, partly because most related studies only cover one glacial cycle. Longer records are available from marine sediment profiles in which the distribution of tephras records frequency changes of explosive arc volcanism with high resolution and time precision. Here we show that tephras of IODP Hole U1437B (northwest Pacific) record a cyclicity of explosive volcanism within the last 1.1 Myr. A spectral analysis of the dataset yields a statistically significant spectral peak at the ~100 kyr period, which dominates the global climate cycles since the Middle Pleistocene. A time-domain analysis of the entire eruption and δ^18^O record of benthic foraminifera as climate/sea level proxy shows that volcanism peaks after the glacial maximum and ∼13 ± 2 kyr before the δ^18^O minimum right at the glacial/interglacial transition. The correlation is especially good for the last 0.7 Myr. For the period 0.7–1.1 Ma, during the Middle Pleistocene Transition (MPT), the correlation is weaker, since the 100 kyr periodicity in the δ^18^O record diminishes, while the tephra record maintains its strong 100 kyr periodicity.

## Introduction

### Volcanism and Climate

There is a range of time scales on which volcanic activity can be affected by internal and external triggers^1^–^4^. For example, Michaut *et al*.^5^ explain periodic variations in activity on the scale of hours at the Soufrière Hills and other volcanoes by formation of gas waves in rising viscous magmas. Volcanic activity can vary on a diurnal to weekly basis influenced by the Earth tides^6^. At the temporal extreme, periods of 10^6^ to 10^7^ years observed in tephra records have been related to geotectonic changes and plate movements (e.g.^7–9^).

Cyclic variations with periods of 10^4^ to 10^5^ years lie in the range of orbital forcings (Milankovitch cycles) of global climate. Marine tephra records show a clear increase in frequency of tephra layers from arc-related explosive eruptions at the onset of the Quaternary glaciations (e.g.^2,3,7,8,10^). Paterne *et al*.^11^ observed a 23 kyr periodicity in the Campanian marine tephra record that appeared related to the Earth’s precession and associated Earth tides. Kutterolf *et al*.^4^ detected a strong 41 kyr periodicity in a Ring of Fire tephra record compiled from drill sites around the Pacific, and associated it with the periodic obliquity changes of the Earth’s rotation axis. Such coincidences of periodic fluctuations in the volcanic eruption frequency with periods of orbitally paced climate changes are presently the strongest evidence that volcanism may be forced by either the orbital parameters themselves (e.g., solid Earth tides) or by their global climatic effects (e.g., continental ice-seawater re-distribution and related sea level change). The increase in frequency of volcanic activity during deglaciation observed over a range of tectonic settings and geographic latitudes (e.g.^12–15^) has been generally attributed to crustal or lithospheric loading and unloading by ice or water masses (e.g.^4,15–18^). Also, unloading by post-glacial erosion can affect continental crust formerly covered by ice shields (cf.^19^). However, the physical processes that link climate changes to volcanism are subject of ongoing discussion.

Important factors for reliable statistical analyses providing a robust link between volcanism and climate are (1) a long and precise chronostratigraphic eruption record and (2) the exact timing of the volcanic frequency change relative to the glaciation cycle, because this timing may place important constraints on the potential physical mechanisms. For example, the peak of hydrothermal activity on the Mid-Atlantic Ridge^20^, Juan de Fuca Ridge^21^ and at the East Pacific Rise^22^ of ∼15 kyr after the glacial sea level fall has been interpreted as possibly due to the delayed response of melt formation and extraction to the hydrostatic pressure minimum associated with the maximum rate of glaciation.

A critical factor in the evaluation of possible orbital periodicities in volcanic records is the length of the record, yet most of the published studies only encompass the last glacial period.

But, are these findings of climate/volcanism interaction also true for earlier glacial periods? The marine tephra record of explosive arc volcanism recovered by IODP Hole U1437B near the Izu-Bonin arc provides such a long well-constrained stratigraphy of time‐varying volcanic activity that spans multiple glacial cycles and offers the opportunity for a crucial test of whether these findings of climate/volcanism interaction are a persistent feature of glacial intervals.

## The Marine Tephra Record of Hole U1437B Izu-Bonin Arc

**Figure 1:**
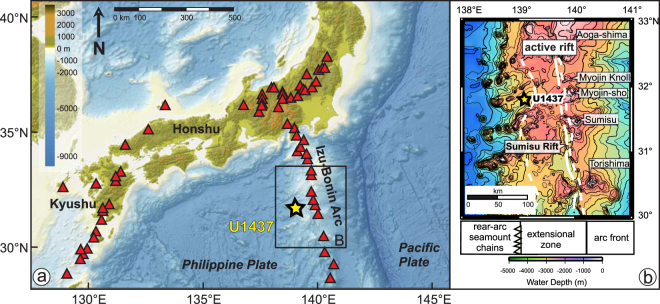
Overview map of Japan and Izu-Bonin arcs (http://www.geomapapp.org; GMRT-Global Multi-Resolution Topography)^46^. Panel (a) includes borehole position of IODP Site U1437 (yellow star). Red triangles mark the locations of active volcanoes along the Japan and Izu-Bonin arcs. (**b**) Bathymetric map with Izu arc front volcanoes and active rift region (extensional zone) and rear-arc seamount chains (modified after^23^ and licensed under CC BY 3.0).

During IODP Expedition 350 IODP Hole U1437B (31.79°N and 139.03°E) was drilled in 2116 m water depth at the Izu reararc in the northwestern Pacific (Fig. 1). Advanced-piston coring (APC) techniques recovered 138 meters (>99% recovery rate) of tuffaceous carbonate mud that is interspersed with numerous dark- and light-colored centimeter to decimeter-thick tephra beds^23^. The sediment record (1) is undisturbed, (2) has high sedimentation rates of ∼12 cm/kyr, (3) has a well characterized and robust age model by δ^18^O stratigraphy and (4) contains 162 tephra layers that represent single explosive volcanic events. Therefore it is very well suited to study the temporal variability of volcanic events over a long time period (∼1.1 Myr) and its temporal relation to glacial cycles.

The ∼1.1 Ma marine tephra inventory of Hole U1437B and the provenance of the tephras are discussed in detail in Schindlbeck *et al*.^24^. The sharp but non-erosive basal contacts, normal grading, and the absence of flow structures (e.g., cross-bedding, imbrication) indicate that the large majority of tephra beds were emplaced as primary (subaerial) fallout rather than from ground-hugging density currents. This is consistent with a quiet sedimentation environment on the flat top of a volcaniclastic apron with no visible redeposition and absence of hiati^23^. The generally high vesicularity and absence of quench textures favor an origin of the discrete tephra layers as ash-cloud fallout that settled through the water column. Most of these ash clouds formed from subaerial eruptions but some may also derive from shallow submarine eruption columns that broke through the water column (cf.^25^). Tephra layer compositions are felsic, mafic or bimodal and have silica contents between 55–78 wt%. Most of the tephra layers (∼80%) derive from the oceanic Izu-Bonin arc volcanic front as confirmed by major and trace element analyses of glass shards^24^ and Sr-Nd isotope ratios, but also widely distributed fallout from the continental Japanese arcs reached the drill site. The majority (>75%) of tephra layers from the Izu-Bonin arc volcanic front show geochemical compositions that are typical for Sumisu (Knolls), Agoashima, Hachijojima, and Torishima volcanoes, with mainly subaerial summits. A subset of tephra layers may originate from the active rift region (<25%) and show geochemical compositions that are typical for Knoll 336, Myojin Rift, and Knolls, Minami Hachijo or South Sumisu^24^. South Sumisu caldera reaches to 300 mbsl^26^ and Myojin Knoll caldera from 500 to 900 mbsl. At the latter Fiske *et al*.^27^ observed up to 200 m thick pumice accumulations and concluded that highly energetic eruptions breached the sea surface and formed subaerial eruption columns. Such conditions, leading to deposits of highly vesicular pumice as observed in Hole U1437B, may be realized for gas-rich silicic magmas erupting at <1000 m water depths (cf.^28^). Considering the present-day high sealevel stand, such eruptions would have been even more likely during past times of low sealevel elevation.

In this contribution we focus on the temporal distribution of the 162 discrete tephra layers across core Sections 350-U1437B-1H to 19F.

## Age Model

The age model, which we use here, is based on δ^18^O data^24^ that was measured on the planktonic foraminifer species (*Neogloboquadrina dutertrei*) selected from successive samples ∼30 cm apart for the upper 30 m (~328 ka), and from samples spaced at ~90 cm in the lower part of Hole U1437B. In total, 43 tie points^24^ were used to correlate the δ^18^O record of Hole U1437B to the LR04 δ^18^O global reference stack^24^. Kars *et al*.^29^ also published an alternative age model that used a slightly different correlation of the same stable oxygen isotope data with LR04 and includes additional nannofossil data. Differences between the two age models are generally quite small, but some ash layer ages at ∼17 mbsf (meters below seafloor) and below 127 mbsf differ by up to 20 kyr (Table S1).

The δ^18^O records of Hole U1437B and the LR04 global reference stack^30^ agree well and yielded an average sedimentation rate of ∼12 cm/kyr that confirm the average sedimentation rate from the shipboard age model^23^. The temporal spacing of the tie points is commonly <30,000 years (less than one glacial/interglacial cycle). Between two successive tie points a linear sedimentation rate is assumed and ages of tephra layers have been calculated by using a linear interpolation. Even if climate changes have caused minor variable sedimentation rates (e.g., variable dust input, biogenic carbonate) within these intervals, the time window between two tie points is too short for such cyclic variations to significantly control the calculated tephra age distribution.

**Figure 2:**
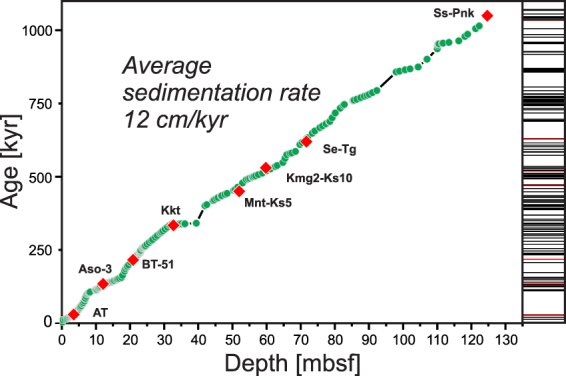
Sedimentation rate calculated from the age model. Green dots are δ^18^O data points^24^ and red diamonds indicate the position of independently dated major Japanese tephras that have been found within the marine tephra record^24^. Each tick of the barcode at the right axis marks one eruptive event. Red tick lines mark the dated Japanese tephras.

The temporal distribution of marine tephra layers in Hole U1437B is represented as a bar code in Fig. 2 with an unit value at the age of each tephra layer.

**Figure 3:**
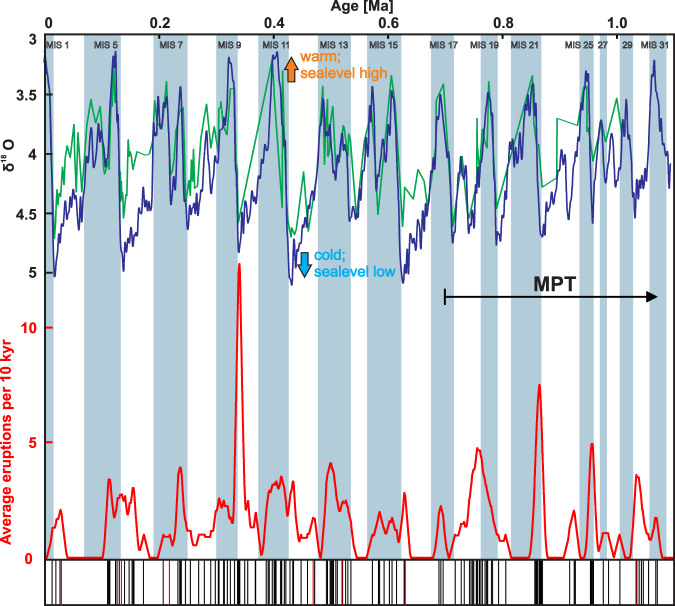
Timeline of the last 1.1 Myr that shows the glacial and interglacial periods and the variation in the volcanic eruption frequency. Upper part: Hole U1437B δ^18^O data^24^ (green line) compared with the LR04 global stack curve of Lisiecki and Raymo^30^ (blue line). Low δ^18^O indicate warm, interglacial periods (gray bars) and high sea level, whereas high δ^18^O values indicate cold, glacial periods (white bars) and low sea level. Lower part: Volcanic eruption frequency variation over the last 1.1 Ma obtained by applying a moving average with a width of 10 kyr (red line) to the ash layer sequence shown as ticks of the barcode at the bottom axis (each tick marks one eruptive event). The red tick lines mark major eruptions from Japan (names are given in Fig. 2). The δ^18^O record of Hole U1437B^24^ was scaled by adding +4 per mil to each value to ease its visual comparison to the global LR04^30^ stack.

The robustness of the δ^18^O age model is supported by two independent observations. The first observation is the Brunhes/Matuyama (C1n/C1R) boundary at 89.95 mbsf (0.786 Ma) and the top of the Jaramillo transition C1r.1n Chron at 119.35 mbsf^23^. Secondly, eight marine tephra layers have been correlated^22^ to widely distributed and independently dated tephras from the Japanese arcs and these ages closely fit those predicted from the age model of Hole U1437B (Fig. 3). The tephra ages range from the 30 ka Aira-Tn (AT) Tephra to the 1.05 Ma Shishimuta-Pink (Ss-Pnk) Tephra and deviate 0.5 to 6.6% from the ages predicted by the age model. We estimate that the error of ash-layer ages is generally less than ±5000 years.

## Temporal Distribution of Tephras and Frequency Analysis

The long time series, densely populated with eruptive events (bottom panel of Fig. 3), allows us to analyze temporal variations of volcanic activity in unprecedented detail. We assume that the eruptions that produced the tephra record are representative for the temporal evolution of the entire volcanism of the respective source regions. To visually highlight periods of decreased and increased volcanic activity a phase-stable 10 kyr running average was applied to this time series (shown as red line in Fig. 3). Persistent cyclic variations with roughly the same amplitude are evident over the entire 1.1 Myr section, with two exceptions: there is an exceptionally high peak in the tephra record at ∼350 ka, and there are unusually few volcanic events recorded for the last 100 kyr.

To investigate a possible causal relationship with glacial cycles, we co-rendered the volcanic eruption time series with the reference global climate proxy δ^18^O^30^ (blue line in Fig. 3) as well as the δ^18^O time series derived from the cores at Hole U1437B (green line in Fig. 3). Bars in Fig. 3 illustrate interglacial (gray) and glacial (white) periods (as defined by the global δ^18^O record). A quasi-periodic cyclicity (100 kyr) is qualitatively visible in the volcanic activity during the entire 1.1 Myr record (Fig. 3).

**Figure 4:**
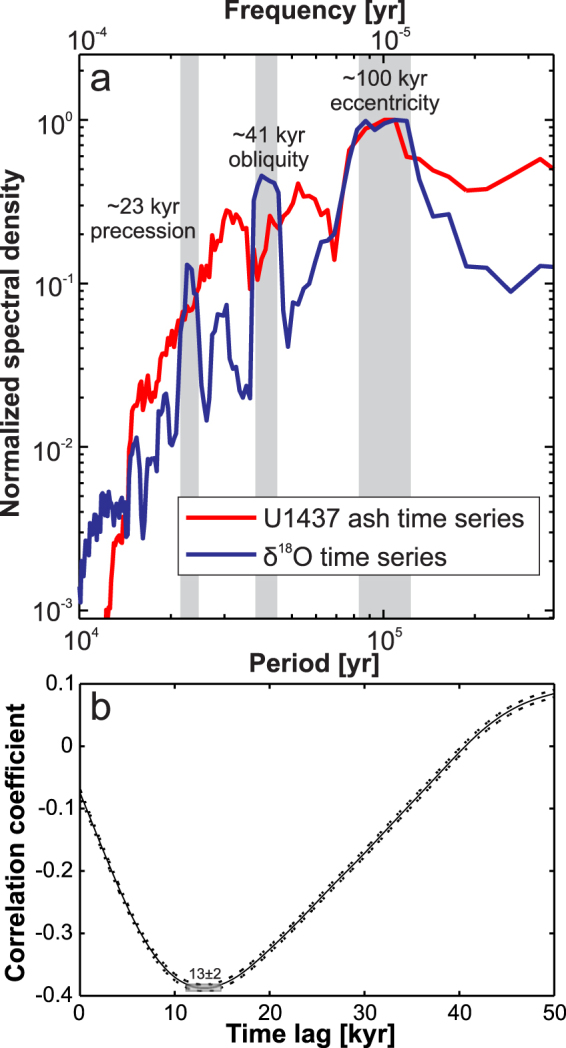
Spectral density and correlation coefficient as a function of lag. (**a**) Comparison of the spectral density of the δ^18^O time series^30^ (blue curve) and the Hole U1437B ash time series (red curve). The spectra are normalized to their maximum value to allow for overlaying of the spectra. An overlapping peak at the 100 kyr period can be observed, which is the Milankovitch eccentricity frequency. Each spectrum has been calculated using multitaper power spectra density estimate with a time bandwidth of 3 and has been normalized to the maximum density value. (**b**) Calculation of the correlation coefficient as a function of lag between the volcanic eruption record of Hole U1437B and the δ^18^O time series^30^. Best correlation with 99% confidence is achieved for a time lag of ∼13 ± 2 kyr between the data sets. The error bars (dots) indicate the 95% confidence limit of the correlation value.

For quantitative assessment of the cyclicity we applied a multi-taper spectral analysis^31,32^ to the tephra and δ^18^O records (Fig. 4a; see supplement for additional information). Just like the δ^18^O record, the volcanic record shows a spectral peak at 100 kyr that is similar to the Milankovitch eccentricity periodicity (Fig. 4a, see als Supplementary Information S1.1). However, other orbital cyclicities with shorter periods (23 ka precession and 41 ka obliquity) evident in the δ^18^O record are not observed in the volcanic record. Instead, peaks occur at around 50 ka and 30 ka, which represent higher harmonics of the pulsed 100 ka signal. While variations at 23 ka and 41 ka may still be present, they are sufficiently weak to be masked by the higher harmonics.

To further investigate the temporal relation of increases in volcanic activity relative to glacial/interglacial transitions we calculated the correlation coefficient as a function of time lag between the δ^18^O time series and the ash time series (Fig. 4b, see also Supplementary Information S1.2), i.e. we shift the entire tephra time series by a certain time lag and calculate the correlation with the unshifted δ^18^O record. Positive lags note a shift of tephra records towards younger times.

A maximum correlation value (−0.39) (normalized covariance) between volcanic eruptions and the global δ^18^O time series^30^ is observed with 99% confidence for a time lag of ∼13 ± 2 kyr between both data sets. This means that if the volcanic time series is shifted by ∼13 ± 2 kyr towards younger times, changes in volcanic eruption frequency are maximal anticorrelated to changes in δ^18^O. The correlations are statistically significant, that is pass the null hypothesis test. A 95% error bar of the correlation coefficient is included in Fig. 4b. The fact that the absolute value of the correlation coefficient is smaller than one indicates that not all variations in eruption frequency can be explained through changes in δ^18^O. This is to be expected due to the fact that other, independent processes influence variations in both δ^18^O and eruption frequency separately. The analysis shows that a maximum eruption activity occurs ∼13 ± 2 kyr before a minimum in δ^18^O, i.e. roughly at the transition from glacial to interglacial. Hence the volcanic activity increases right within the time interval of the fastest change from the δ^18^O maximum (sea level low stand) to the δ^18^O minimum (sea level high stand) also known as glacial termination.

In order to test the robustness of our results particularly with respect to age uncertainties, we repeated the above calculation on two additional tephra time series. The first of these is built by using the alternative age model for Hole U1437 proposed by Kars *et al*.^29^. For the second test we added a random value between of ±5 kyr (estimated age error) to each of the original tephra ages to mimic tephra age uncertainties. The results are shown and compared in the Figure S2. The important result of the comparison is that the spectral density of the 100 kyr periodicity remains the same, and the correlation coefficient versus time lag function for this periodicity varies little between −0.39 to −0.33 and 13 ± 2 to 12 ± 2 kyr.

The age differences between the three series do, however, affect the higher frequencies. The 30 and 50 kyr periods seen in the original record (Fig. 4a) no longer appear, or are replaced by other periods, in the analyses of the two other time series (Fig. S2). This is why we do not further discuss possible higher frequency variations but rather focus on the robust 100 kyr cyclicity.

### Time-dependent frequency analysis

So far we investigated the correlation of volcanic and δ^18^O time series over the entire 1.1 Ma age range of Hole U1437B. However, it is well known that the pattern of the δ^18^O time series changes with time. Prior to 1.25 Ma, it is dominated by the 41 kyr periodicity while at times <0.7 Ma the 100 kyr periodicity vastly dominates. The time interval 0.7 to 1.25 Ma is characterized by a gradual transition from one pattern to the other and is called the Middle Pleistocene Transition (MPT; Fig. 3), a period of higher frequency, non-periodic glacial cycles^33^.

**Figure 5:**
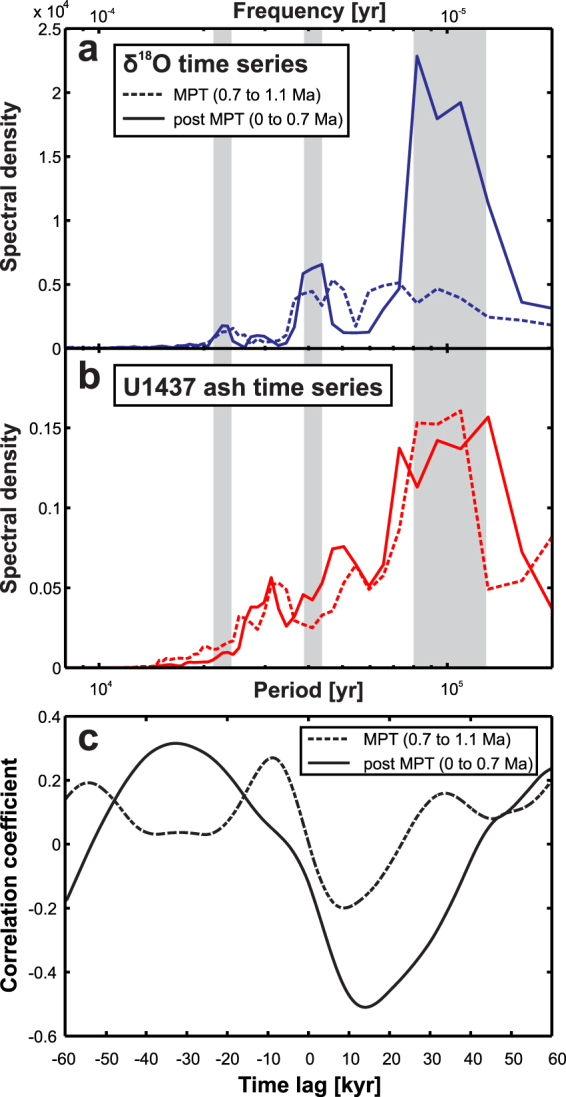
Time dependent spectral density and correlation coefficient as a function of lag. (**a**) Spectral characteristics of the δ^18^O record (LR04)^30^ and (**b**) spectral characteristics of the volcanic eruption record during the MPT (red line) and post-MPT (blue line). Gray bars are the same as in Fig. 4a. (**c**) Correlation coefficient between the δ^18^O and eruption records during the MPT (red line) and post-MPT (blue line). The correlation coefficient during the MPT is relatively small for all time lags and shows a complicated behavior, whereas the post-MPT correlation coefficient has a maximum of −0.51 at a time lag of ∼13 kyr.

In order to investigate how the correlation with the volcanic record is affected by these changes in the δ^18^O record, we divide our time series into two sections, a MPT section from 0.7 to 1.1 Ma and a post-MPT section from 0 to 0.7 Ma. While the spectral characteristics of the global δ^18^O record change quite dramatically from predominantly high-frequency content during the MPT to low-frequency content post-MPT (Fig. 5a), the spectral characteristics for the volcanic eruption record show a continuous predominantly low-frequency content (Fig. 5b). Consequently, the correlation between the δ^18^O and eruption records is poor for all time lags during the MPT (Fig. 5c). In contrast, post-MPT the correlation coefficient becomes even better (−0.51) and yields a similar time lag of ∼13 kyr compared to the above analysis across the entire age range. Thus, we conclude that the observed correlation over the entire record is mainly due to a pronounced correlation post-MPT, which masks the poor correlation during MPT.

## Discussion

### Significance of the tephra record

The general assumption in the interpretation of correlations between climate and volcanic signals is that large climate changes (continental ice sheet-seawater redistribution, and mass transport from glacial continental erosion) provoke changes in volcanic activity (e.g.^4,8,11,15,34^). The climate changes in turn are thought to be partly controlled by orbital parameters (e.g.^35–37^), which determine the insolation intensity.

Previous studies that encompass data of volcanic activity over a longer period of time observed periods of increased volcanism on different scales that might be associated with the Milankovitch cycles. Our present 1.1 Ma tephra record has a high spectral density peak for 100 kyr, but fails to produce significant spectral densities for the 23 and 41 kyr periods. One fundamental difference is that the Hole U1437B tephra record is from one site, whereas the Kutterolf *et al*.^4^ tephra record had been compiled from multiple sites around the Pacific. The compiled record contains contributions from mainly continental arcs, whereas our record is dominated by an oceanic arc. Local geological controls probably modulate the climatic effects on volcanism (e.g.^12,14,38^). However, the more important difference may be that the compiled record contains contributions from a wide range of latitudes. Location latitude may play a crucial role as volcanism at sites closer to the ice sheets may react differently from near-equatorial volcanism (e.g.^38,39^).

For example, a commonly invoked physical interpretation of the effect of global climate changes on volcanism is that unloading, i.e. pressure release, favors enhanced melting in the mantle source as well as the creation of lithospheric pathways through extension (e.g.^12,16^). Modeling by Schmidt *et al*.^40^ suggests that the magnitude of pressure drop during deglaciation would be sufficient to cause significant increase in mantle melt production. Consequently, while ice unloading would enhance volcanism at high latitudes, elevated sea level would reduce volcanism at low latitudes.

Our volcanic record from a mid-latitude (32° N) oceanic setting reveals the 100 kyr periodicity that involves the by far largest ice-sea water redistributions. Our data show that the maximum eruption frequency occurs right during the time when deglaciation generates the fastest rate of pressure change. The melting interval (from maximum to minimum δ^18^O; Fig. 3) only lasts roughly 20 kyr compared to the roughly 80 kyr ice build-up; hence the time lag of ~13 kyr before δ^18^O minimum means a time lag of approximately 7 kyr after the glacial maximum. During this time deglaciating continents experience a high rate of pressure release while the ocean floor experiences a high rate of pressure increase with rising sea level.

With most ash layers in our record originating at the Izu-Bonin arc, it is clear that the increase in volcanism ~7 kyr after glacial maximum cannot be explained by the simple pressure-release model. Considering glaciation on the Japanese mainland (e.g.^41^), we speculate that deglaciation at Japan and sea level rise offshore caused a change in the stress balance across the subduction system, which favored an increase in explosive volcanism. However, this hypothesis needs to be tested by dedicated modeling and further investigations.

Obviously there is a need for more comprehensive volcanic records to investigate how tectonic setting, latitude and nature of the record affect the frequency contents that can be observed.

### Significance of MPT and post-MPT tephra frequencies

The very good correlation between tephra and δ^18^O records (sea level proxy) for the 100 kyr periodicity during post-MPT times (Fig. 5c) provides a strong argument for a causal link between the deglaciation of large ice sheets and an increase in volcanic activity. But does the poor correlation during the MPT mean that such a causal link did not exist although the strength of the 100 kyr signal in the tephra record is maintained? The δ^18^O record for the time span 0.7 to 1.1 Ma does, in fact, contain a 100 kyr periodicity, but its spectral density is relatively low because of complex interferences between orbitally controlled insolation and terrestrial parameters such as weathering rates, atmospheric CO_2_ budget and sea-ice distribution that strengthens other periodicities (e.g.^33,42^). The 3 Myr δ^18^O record compiled by Bintanja and van de Wal^43^ shows that larger amplitude excursions at a 100 kyrs periodicity already occur since about 1 Ma, and their modeling results suggest that this is due to the formation of large northern hemisphere ice sheets since that time. Sosdian and Rosenthal^44^ use foraminifer Mg/Ca ratios to separate the effects of ocean bottom water temperature and continental ice sheet volume on the 3 Myr δ^18^O record; they found that cooling and amplitude increase precede the major frequency shift and were associated with more ice formation per degree of cooling in the northern hemisphere. This work confirms and refines the earlier work by Mudelsee and Schulz^45^ who already noted that the ice-volume increase preceded the change to the dominant 100 kyr cycle in the δ^18^O record. Both empirical and modeling studies remain somewhat uncertain on when the Mid-Pleistocene large ice sheets really began, but it seems clear that they well pre-dated the 0.7 Ma onset of post-MPT conditions. If we accept that our observed post-MPT correlation between tephra and δ^18^O records implies that partial melting of huge ice sheets triggered an increase in volcanism at a 100 kyr periodicity, and as we see that periodicity maintained in the tephra record up to 1.1 Ma, then it seems reasonable to conclude that the same triggering mechanism operated over the entire age range. This would imply that large northern hemisphere ice sheets already existed since about 1.1 Ma. Another consequence then is that a poor correlation in spectral analysis does not necessarily eliminate the existence of a causal link, but that it is contaminated by changes in variations at different frequencies in the δ^18^O records.

## Conclusion

A spectral analysis of the 1.1 Myr marine tephra record of Hole U1437B in the northwestern Pacific yields a statistically significant spectral peak at the ~100 kyr period. Correlation between the tephra record and the δ^18^O record as climate/sea level proxy shows that volcanism peaks after the glacial maximum and ∼13 ± 2 kyr before the δ^18^O minimum. The correlation is especially good for the last 0.7 Myr after the Middle Pleistocene Transition, whereas the correlation between 0.7 and 1.1 Ma during the MPT is relatively poor because then the 100 kyr signal, which is maintained strong in the tephra record, is comparatively weak in the δ^18^O record. This means that the observed correlation over the entire record is strongly dominated by the good correlation post-MPT. The observed correlation strongly suggests that partial melting of the large ice sheets triggered an increase in volcanism. The persistence of the strong 100 kyr periodicity in the tephra record well into MPT times suggests that this causal link also persisted although the 100 kyr periodicity appears only weakly in the δ^18^O spectrum. This agrees with investigations implying the formation of large northern hemisphere ice sheets well before the end of the MPT.

## Electronic supplementary material

Supplementary Information

Table S1
