# Supplementary material for: 100- kyr cyclicity in volcanic ash emplacement: evidence from a 1.1 Myr tephra record from the NW Pacific
Source: Sci Rep. 2018 Mar 13;8:4440. doi: 10.1038/s41598-018-22595-0 (PMC5849666; doi:10.1038/s41598-018-22595-0)
Supplement: Supplementary file 1 — Supplementary Information [file 41598_2018_22595_MOESM1_ESM.pdf]

100- kyr cyclicity in volcanic ash emplacement: evidence from a 1.1 Myr tephra record from the NW Pacific

Julie C. Schindlbeck[1,2*], Marion Jegen[1], Armin Freundt[1], Steffen Kutterolf[1], Susanne M. Straub[3], Maryline J. Mleneck-Vautravers[4], Jerry McManus[3]

[1] GEOMAR Helmholtz Centre for Ocean Research Kiel, 24148 Kiel, Germany

[2] Institute of Earth Sciences, Heidelberg University, Im Neuenheimer Feld 234-237, 69120 Heidelberg, Germany

[3] Lamont-Doherty Earth Observatory of Columbia University, Palisades, New York 10964, United States

[4] Department of Earth Sciences, University of Cambridge, Downing Street, Cambridge CB26 3EQ, United Kingdom

*Corresponding author email address: Julie.Schindlbeck@geow.uni-heidelberg.de

S1 Time series analysis

The time series of volcanic eruptions and $\delta^{18}O$ have been analysed in the time as well as frequency domain. Both analyses are described in more technical detail below.

S1.1 Spectral Analysis of $\delta^{18}O$ *and Tephra Record*

Using spectral analysis we determine the distribution of power of the time series into frequency components (power spectra). The spectral analysis was performed based on the multi-taper spectral analysis method[31,32]. We use the multi taper method since it reduces estimation bias in the power spectra. It estimates the spectral density for each frequency by obtaining multiple independent estimates from the same time series using a sequence of discrete prolate spheroidal (DPSS) tapers. The resulting spectral resolution is determined by the time-bandwidth product. In our analysis we use a medium value of the time bandwidth product of 3 in order to balance resolution

and smoothness of the spectrum. The methodology also allows for the determination of a confidence level in the spectra.

Figure S1 shows the spectra derived for the $\delta^{18}O$ and tephra records, normalized by the maximum power of each spectrum, together with 95% and 67% confidence levels, which correspond to two-sigma and one-sigma standard deviations. The spectra are depicted in a log-linear time scale for better visibility. For both time series a peak at 100 ka is distinct from other peaks. The peaks of the tephra time series centred at periods of 50 ka and 33 ka constitute higher harmonics caused by the pulsed 100 ka signal, and could potentially mask a smaller peak at 40 ka. As a reference for the relevance of the 100 ka peak in the tephra sequence, we also show the spectral density with 1-sigma standard deviation for an artificial random tephra sequence.

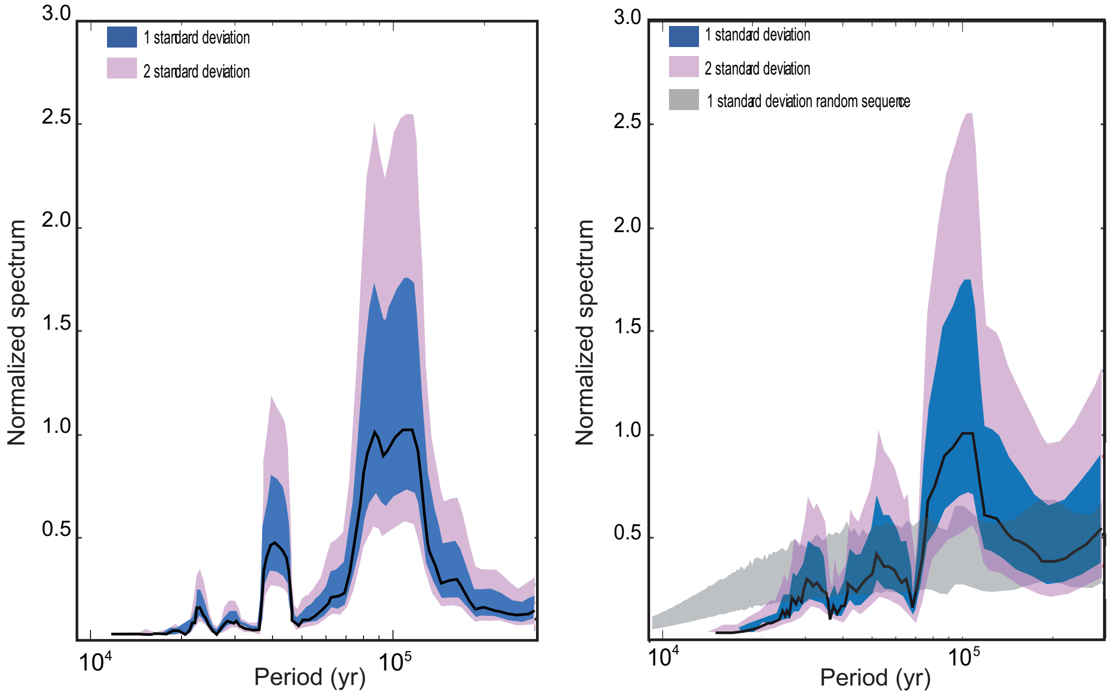

Figure S1. Spectra derived for the $\delta^{18}O$ (left panel) and tephra records (right panel), normalized by the maximum power of each spectrum, together with 95% and 67% confidence levels, which correspond to two-sigma and one-sigma standard deviations (blue and pink shades).

S1.2 Time Domain Analysis

The first step of the analysis of the tephra time series in the time domain consisted of applying a 10,000 year running average to the tephra time series. This procedure entails that at each time increment (10 years) of the tephra time series, volcanic eruptions within a window of 5,000 years prior and after this point of time are averaged and assigned to that point of time. To minimize a possible phase shift induced by the filter, the filter is applied also in the reverse direction. In the spectral domain, the filter acts as a low pass filter, supressing spectral energies with periods <10,000 years.

The filtered tephra series were used to calculate the cross correlation with the $d^{18}O$ time series. The correlation coefficient is a statistical measure of a linear relationship between two time series. In the context of our time series we use it to determine whether variations in the $\delta^{18}O$ time series are related to variations in eruption frequency. The correlation coefficient reaches maximum positive /minimum negative values, if variations in the time series are similar, aligned in time and have the same/opposite sign. Shifting one harmonic time series with a particular period in time with respect to another similar harmonic time series changes the correlation coefficient. For a time shift (lag) at which the principal variations are aligned positively, the correlation coefficient reaches a maximum value. A further lag by half of the fundamental period would align the variations with opposite sense and result in a minimum negative value.

Absolute values of the correlation coefficient indicate how well variations in one time series can be explained by a linear model through variations in the other time series. The correlation coefficient may reach 1/-1 if an exact linear relationship between the variations exists. Lower values may be attributed to the fact that the relationship is not exactly linear or that only part of the variations (for example. variations with particular periods) are linearly related or to uncorrelated noise on the time series. To assess whether these lower correlation values are yet statistically significant, we additionally calculate a probability that the given correlation coefficient is reached by chance if the true correlation is zero. Probabilities smaller than 5% indicate that this is not the case.

In our analysis, a positive lag indicates that the tephra time series is shifted towards older times. We observe an extreme value of the correlation, calculated of the entire length of time series, of -0.39 for a lag of 13 kyr. At a time lag of 13 kyr, $\delta^{18}O$ and eruption frequency exhibit aligned variations with opposite signs. An increase in eruption frequency thus follows a minimum in $\delta^{18}O$ after 13 kyr. The negative correlation is stronger (-0.51), if only the post-MPT segments of the time series are considered, while during the MPT the correlation is weaker (-0.19). The finding suggests that the correlation over the entire time series is dominated by post-MPT correlations. The probabilities that any of the correlation values stated above are reached by chance, are smaller than 0.1 %. The correlation coefficient analysis thus indicates that not all yet part of the variations in eruption frequency, particularly in the post-MPT phase, can be statistically significantly correlated to changes in $\delta^{18}O$.

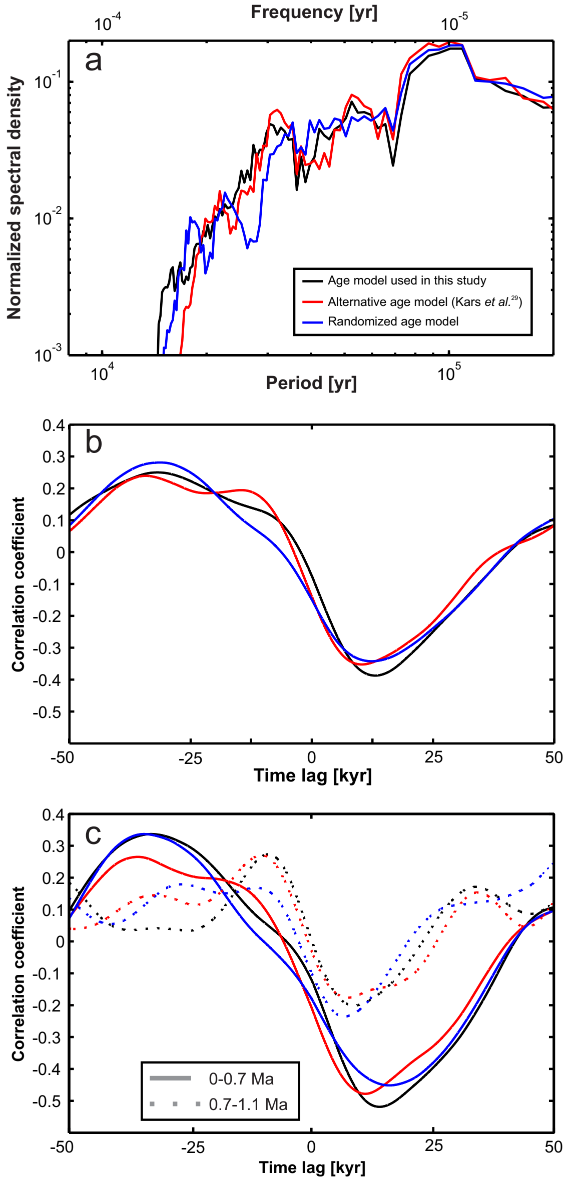

Figure S2. Comparison of the spectral analysis results for the three different tephra time series explained in the text. a) All three ash time series share the strong peak at 100 kyr, which is the Milankovitch eccentricity frequency. b) The correlation coefficients and related time lags between the respective tephra records of Hole U1437B and the $\delta^{18}O$ time series[30] are very similar for the three cases. c) This is also true when the correlation coefficients are determined separately during the MPT (0.7-1 Ma) and post-MPT (0.7-1.1 Ma). The correlation coefficient during the MPT is relatively small for all time lags and shows a complicated behavior, whereas the post-MPT correlation coefficients have maximum values between 10 and 13 kyr.
